# Pan-Genome and Transcriptome-Guided Analysis Reveals Duplication-Driven Evolution and Candidate MYB–bHLH Modules Associated with Fruit Development in Pear

**DOI:** 10.3390/plants15131961

**Published:** 2026-06-25

**Authors:** Guoming Wang, Nan Zhu, Xun Sun, Kaijie Qi, Zhihua Guo

**Affiliations:** 1 Jiangsu Key Laboratory for Conservation and Utilization of Plant Resources, Institute of Botany, Jiangsu Province and Chinese Academy of Sciences, Nanjing 210014, China; wangguoming@jib.ac.cn; 2 Jiangsu Engineering Research Center for Pear, College of Horticulture, Nanjing Agricultural University, Nanjing 210095, China; nnz916@zju.edu.cn (N.Z.); sunxun1991@njau.edu.cn (X.S.); qikaijie@njau.edu.cn (K.Q.); 3 College of Agriculture, Shihezi University, Shihezi 832003, China

**Keywords:** pear, bHLH, MYB, pan-genome, fruit development

## Abstract

Gene duplication and subsequent selection are central to genome evolution and transcription factor diversification, but the conservation and divergence of the basic helix–loop–helix (bHLH) family in pear remain unclear from a pan-genome perspective. Here, we performed a pan-genome and transcriptome-guided analysis across 15 pear genome assemblies, including Asian pear, European pear, and hybrid/haplotype assemblies. Genome-wide duplicated gene pairs were classified into different duplication types, and Ka, Ks, and Ka/Ks values were calculated to establish an evolutionary background for duplicated pear genes. Based on this framework, 3222 *bHLH* were identified and grouped into evolutionary clades and orthologous gene groups. The pear bHLH family contained conserved core members and variable dispensable members, indicating both functional conservation and genome diversification. Duplication and Ka/Ks analyses showed that WGD/segmental duplication contributed to bHLH expansion and that most duplicated *PbrbHLH* gene pairs were constrained by purifying selection. By integrating 17-tissue and fruit-development transcriptomes from three pear cultivars, 39 fruit-development-associated PbrbHLHs were selected. Co-expression analysis with 185 PbrMYBs identified candidate MYB–bHLH co-expression modules from the available pear fruit-development transcriptomes. These results provide an evolutionary framework for pear bHLH diversification and candidate regulatory modules for future functional studies.

## 1. Introduction

Pan-genomes have become increasingly important for describing genetic diversity beyond a single reference genome, supported by the growing availability of high-quality plant genome assemblies. A pan-genome integrates genomic information from multiple accessions or cultivars and can capture core and variable genomic components, including gene presence/absence variation, copy-number variation, and structural variation [1,2]. So, pan-genome analysis provides a broader framework for studying genome evolution, domestication, adaptation, and trait-related genetic variation in plants. Pan-genome resources are also useful for gene-family characterization. Most traditional gene-family studies are based on a single reference genome, which may miss accession-specific members or underestimate copy-number differences within a species. By comparing OGGSs across multiple genomes, pan-genome-based analysis can distinguish core, softcore, dispensable, and private members of a gene family and provide a more complete view of its evolutionary history [3,4]. Therefore, pan-genome analysis highlights the value of this strategy for large plant gene families.

Pear (*Pyrus*) is an important temperate fruit crop with abundant genetic diversity and a long cultivation history [5]. Cultivated pears mainly include Asian pears, European pears, and interspecific or intergroup hybrids, with representative cultivated species such as *Pyrus pyrifolia*, *Pyrus bretschneideri*, *Pyrus ussuriensis*, *Pyrus sinkiangensis*, and *Pyrus communis* [6]. These pear species and cultivars differ substantially in fruit size, texture, ripening behavior, flavor, and stress adaptation, providing valuable genetic resources for fruit biology and breeding. In recent years, the release of multiple high-quality pear genome assemblies has provided an opportunity to study gene-family evolution from a broader genomic perspective [6]. Compared with single-reference-based analyses, a pan-genome framework can better capture conserved and variable gene members among pear germplasm, making it useful for investigating gene duplication, retention, loss, and potential functional divergence in important transcription factor families.

The basic helix–loop–helix (bHLH) transcription factor family is one of the largest transcription factor families in plants and plays broad roles in plant growth, development, and environmental responses [7,8]. More than 65 plant bHLH family studies had been reported by May 2024, covering 53 species from different orders and families, indicating the broad interest in this gene family and its evolutionary diversity [4]. bHLH proteins are characterized by a conserved bHLH domain, in which the basic region is generally associated with DNA binding, whereas the helix–loop–helix region contributes to dimerization and protein–protein interactions [9]. Previous studies have shown that plant bHLHs can function as transcriptional activators or repressors and participate in diverse biological processes, including cell fate determination, stomatal and root hair development, iron homeostasis, light and temperature responses, fruit development, specialized metabolism, and responses to abiotic and biotic stresses [8]. These diverse functions suggest that the expansion and diversification of *bHLH* genes may have contributed to the evolution of complex regulatory networks in plants.

bHLH proteins often act through combinatorial transcriptional modules with other regulatory proteins. The MYB–bHLH–WD40 (MBW) complex is a classical regulatory system involved in anthocyanin and proanthocyanidin biosynthesis, flavonoid metabolism, and epidermal cell differentiation [10]. In this complex, MYB proteins generally provide target specificity, bHLH proteins contribute to partner interaction and transcriptional regulation, and WD40 proteins help stabilize the regulatory complex [11]. Structural studies further showed that MYB and bHLH transcription factors can form specific protein complexes through defined interaction modes, suggesting that MYB–bHLH combinations may act as important regulatory units in plant development and metabolism [12,13]. These findings suggest that MYB–bHLH combinations may represent important regulatory units linking transcription factor family evolution with developmental and metabolic regulation in plants. Therefore, integrating bHLH family evolution with MYB–bHLH co-expression analysis may help identify candidate regulatory modules associated with pear fruit development.

Pear fruit development and ripening are complex biological processes that determine important agronomic and quality traits, including fruit size, texture, firmness, flavor, sugar and acid accumulation, and coloration. Transcriptome studies across major cultivated pear species have revealed extensive gene expression reprogramming during fruit development and maturation, suggesting that transcriptional regulation is closely associated with the formation of fruit developmental and quality traits [14,15,16]. In particular, several transcription factor families have been implicated in fruit-related processes. For example, MYB transcription factors have been associated with flavonoid and anthocyanin metabolism [17,18,19,20], while bHLH members such as PbbHLH164 have been reported to participate in ethylene biosynthesis and fruit ripening regulation [21]. These findings indicate that transcription factors may act either independently or in combination to regulate different aspects of pear fruit development. However, the evolutionary features of pear *bHLH* genes in a pan-genome context and their potential association with *MYB* genes during fruit development remain insufficiently understood.

Although the *bHLH* gene family has been investigated in pear [22,23], their conservation, diversification, and duplication-driven evolution in the pear pan-genome remain poorly understood. Compared with previous single-reference-based pear bHLH studies, the pan-genome strategy used here allows a broader evaluation of *bHLH* gene conservation, presence/absence variation, and copy-number variation across multiple pear genome assemblies. Moreover, the potential association between PbrbHLHs and MYB-related regulatory modules during pear fruit development has not been systematically explored. In this study, we identified *bHLH* genes from 15 pear genome assemblies and analyzed their chromosomal distribution, OGG classification, pan/core composition, evolutionary relationships, duplication patterns, and selection pressure. By integrating tissue and available fruit-development transcriptome datasets, we further screened candidate PbrbHLHs and constructed a candidate PbrMYB–PbrbHLH co-expression network. The 15 genome assemblies were used for pan-genome-level characterization, duplication analysis, and selection analysis of the pear bHLH family, whereas the transcriptome datasets were used for candidate gene prioritization and expression-based support. This study provides new insights into the pan-genome evolution of pear *bHLH* genes and identifies candidate MYB–bHLH co-expression modules for future functional studies of pear fruit development.

## 2. Results

### 2.1. Genome-Wide Identification and Chromosomal Distribution of Pear bHLH Genes

**Figure 1:**
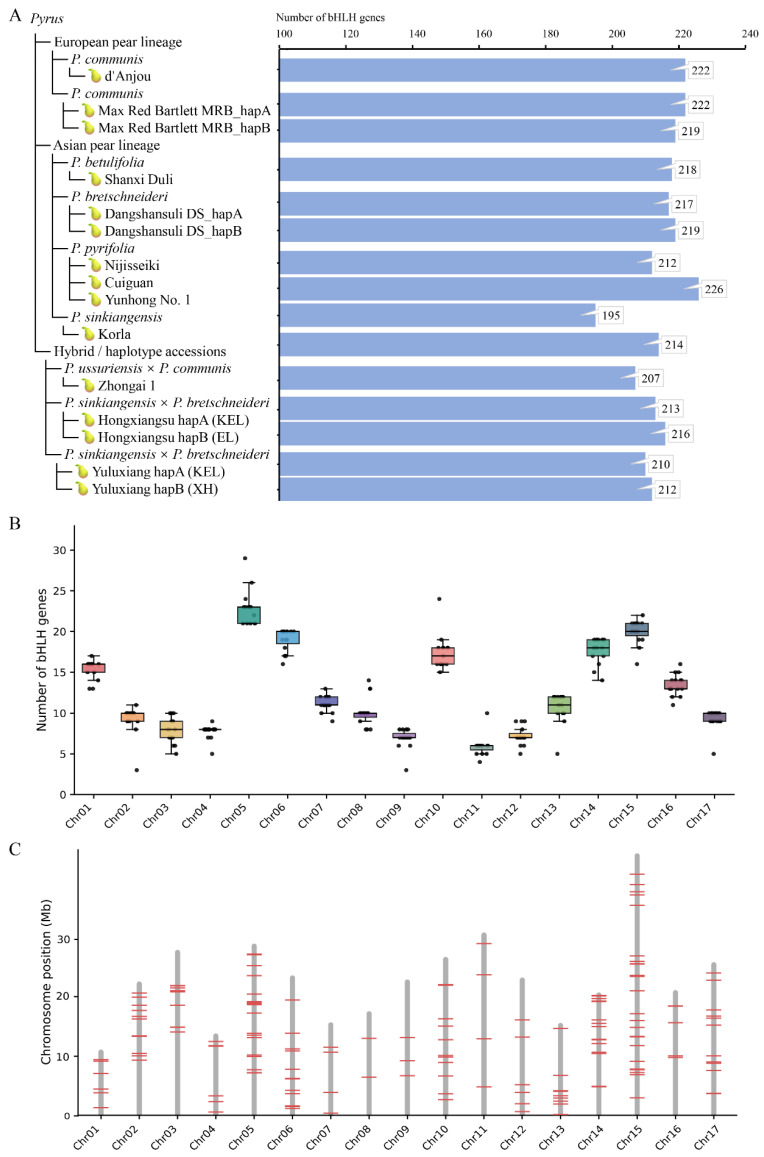
Genome-wide identification and chromosomal distribution of *bHLH* genes in pear. (**A**) Taxonomic and haplotype framework of the 15 pear genome assemblies used in this study, together with the number of *bHLH* genes identified in each assembly. The analyzed assemblies include European pear, Asian pear, and hybrid or haplotype-resolved pear accessions. (**B**) Chromosome-wise distribution of *bHLH* gene numbers across the 15 pear genome assemblies. Each dot represents one assembly, and boxplots show the distribution of *bHLH* gene numbers on each chromosome. (**C**) Chromosomal localization of *bHLH* genes in the ‘Dangshansuli’ reference genome. Grey vertical bars represent chromosomes, and red horizontal marks indicate the physical positions of *bHLH* genes. Chromosome positions are shown in megabases (Mb).

To systematically characterize the *bHLH* gene family in pear, protein sequences from 15 pear genome assemblies were used for genome-wide identification. These assemblies included European pear, Asian pear, and several hybrid or haplotype-resolved accessions, including *P. communis*, *P. betulifolia*, *P. bretschneideri*, *P. pyrifolia*, *P. sinkiangensis*, and hybrid-derived materials (Figure 1A and Table S1). After candidate screening and PF00010 domain confirmation, a total of 3222 *bHLH* genes were identified from the 15 pear genome assemblies, with the number of *bHLH* genes in individual assemblies ranging from 195 to 226 (Figure 1A and Table S2). ‘Cuiguan’ contained the largest number of *bHLH* genes, with 226 members, whereas ‘Yunhong No. 1’ contained the fewest, with 195 members. The haplotype-resolved assemblies generally showed comparable *bHLH* gene numbers within the same accession, such as DS_hapA and DS_hapB with 217 and 219 genes, MRB_hapA and MRB_hapB with 222 and 219 genes, ‘Hongxiangsu’ hapA and hapB with 213 and 216 genes, and ‘Yulixiang’ hapA and hapB with 210 and 212 genes, respectively (Figure 1A). These results indicate that the bHLH family size is broadly conserved among the analyzed pear assemblies, although moderate variation exists among cultivars and haplotypes. The chromosome-wise distribution of *bHLH* genes was further examined across the 15 assemblies. *bHLH* genes were detected on all 17 chromosomes, but their distribution was uneven (Figure 1B). Several chromosomes, especially Chr05, Chr06, Chr10, Chr14, and Chr15, generally carried relatively more bHLH members, whereas Chr04, Chr09, Chr11, and Chr12 tended to contain fewer members. This pattern suggests that the chromosomal distribution of *bHLH* genes is not random and that certain chromosomes contribute more strongly to bHLH family expansion across pear genomes. Using the ‘Dangshansuli’ reference genome as a representative, the physical positions of *bHLH* genes were mapped onto the 17 chromosomes (Figure 1C). The mapped genes were distributed across all chromosomes, with several regions showing locally dense *bHLH* gene distribution, particularly on chromosomes with higher gene numbers. This chromosomal localization provides a reference framework for subsequent orthogroup classification, copy-number variation analysis, and Pbr ID-based functional annotation of pear *bHLH* genes.

### 2.2. Pan-Genome Classification and Copy-Number Variation of Pear bHLH OGGs

**Figure 2:**
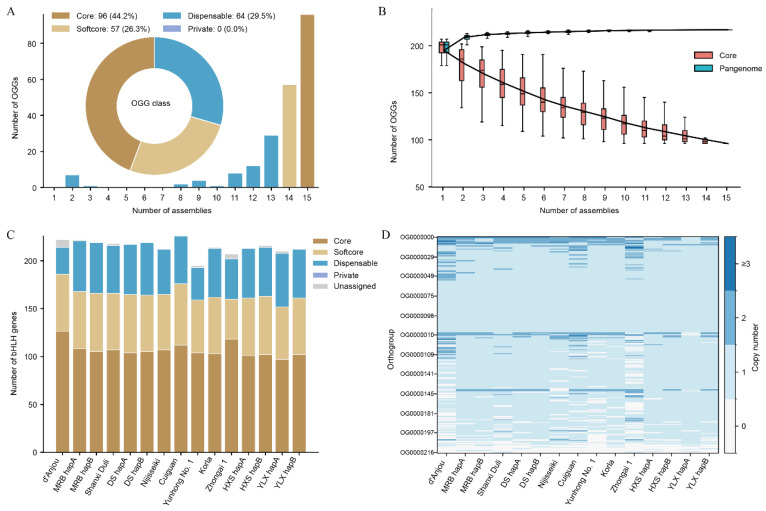
Pan-genome classification and copy-number variation of pear bHLH orthogroups. (**A**) Distribution of bHLH orthogroups (OGGs) according to the number of genome assemblies in which they were detected. The outer bar plot shows the number of OGGs present in different numbers of assemblies, and the inset donut chart summarizes the proportions of core, softcore, dispensable, and private OGGs. (**B**) Pan/core curve of bHLH OGGs with increasing genome number. The pangenome curve represents the cumulative number of OGGs detected as more assemblies were included, whereas the core curve represents the number of OGGs shared by the included assemblies. (**C**) Gene-level composition of bHLH members in each assembly based on OGG classification. Different colors indicate genes assigned to core, softcore, dispensable, private, or unassigned categories. (**D**) Copy-number variation heatmap of bHLH OGGs across the 15 pear genome assemblies. Colors represent copy number, with values of three or more copies grouped as ≥3. MRB, ‘Max Red Bartlett’; DS, ‘Dangshansuli’; HXS, ‘Hongxiangsu’; YLX, ‘Yuluxiang’.

To further characterize the conservation and variation of the pear bHLH family across the 15 genome assemblies, the identified *bHLH* genes were clustered into orthogroups. In total, 217 bHLH OGGs were obtained, including 96 core OGGs, 57 softcore OGGs, and 64 dispensable OGGs, accounting for 44.2%, 26.3%, and 29.5% of all OGGs, respectively (Figure 2A and Table S3). No private OGG was detected in this analysis. The distribution of OGG presence frequency showed that most OGGs were shared by many assemblies, with the largest proportion detected in all 15 assemblies. This pattern indicates that the pear bHLH family is generally conserved across the analyzed genomes, although a subset of OGGs showed presence/absence variation among different assemblies. The pan/core curve further supported this pattern. As the number of included genome assemblies increased, the bHLH pangenome expanded only slightly and gradually approached saturation, whereas the number of core OGGs decreased and then tended to stabilize (Figure 2B). When all 15 assemblies were included, the pangenome contained 217 OGGs, while 96 OGGs were retained as core OGGs. This relatively limited increase in the pangenome curve suggests that additional pear assemblies contributed only a small number of newly detected bHLH OGGs, consistent with the conserved nature of this transcription factor family. At the gene level, the composition of bHLH members varied moderately among assemblies (Figure 2C). Core and softcore genes accounted for the major proportion of bHLH members in each assembly, while dispensable genes contributed to the differences in family size among assemblies. Only a small number of genes were not assigned to any OGG and were classified as unassigned genes. These results suggest that the variation in *bHLH* gene number among pear assemblies is mainly associated with changes in dispensable OGGs and limited unassigned members. The copy-number heatmap provided a more detailed view of OGG-level variations across assemblies (Figure 2D). Many OGGs showed conserved presence patterns among the 15 assemblies, while several OGGs displayed copy-number differences or absence in specific assemblies. Some OGGs contained multiple copies in particular genomes, indicating lineage- or haplotype-associated expansion of certain bHLH members. Together, these results show that the pear bHLH family is broadly conserved at the orthogroup level, with moderate variation mainly reflected in dispensable OGGs and copy-number differences among assemblies.

### 2.3. Evolutionary Classification of Pear bHLH OGGs

**Figure 3:**
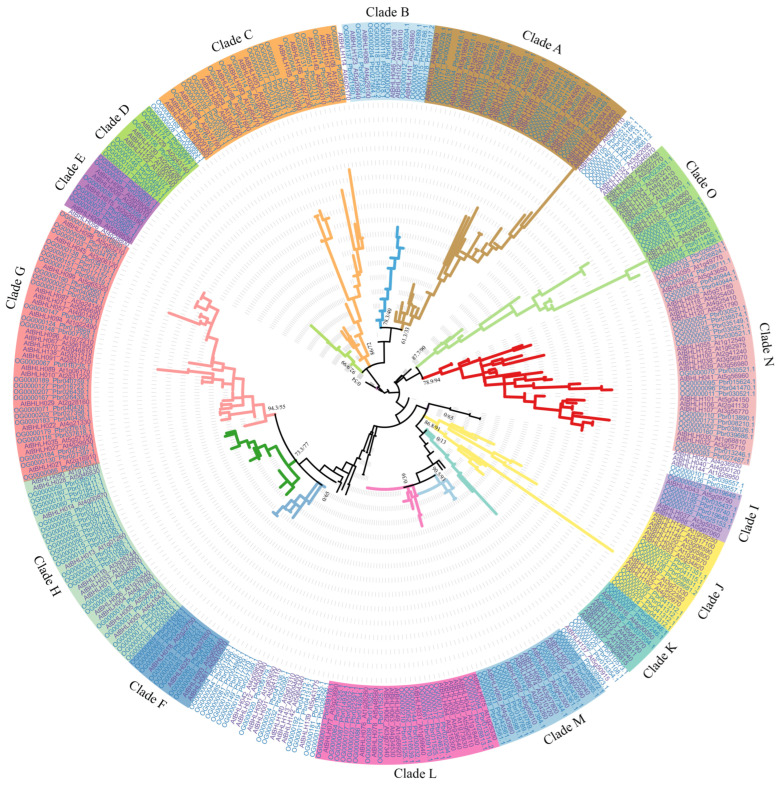
Evolutionary classification of pear bHLH OGGs with *Arabidopsis* bHLH reference proteins. The evolutionary tree was constructed using *Arabidopsis* bHLH proteins and representative pear bHLH OGG sequences. Major evolutionary groups were designated Clade A–O. Support values for the major clades are shown as SH-aLRT/UFBoot. Blue font indicates representative pear bHLH OGG sequences, whereas purple font indicates *Arabidopsis* bHLH reference proteins.

To classify pear bHLH orthogroups in an evolutionary context, a tree was constructed using representative pear bHLH OGG sequences and *Arabidopsis* bHLH reference proteins. Based on the topology of the tree, the analyzed bHLH sequences were grouped into 15 major clades, designated Clade A to Clade O (Figure 3 and Table S3). A small number of sequences were located outside the major Clade A–O groups or on weakly supported branches; these sequences were retained in the OGG annotation table but were not assigned to major clade-specific categories. The number of pear OGG representatives differed among clades. Clade G contained the largest number of pear bHLH OGGs, followed by Clade H, Clade N, Clade A, Clade C, and Clade L. In contrast, Clade K and Clade I contained relatively few pear OGGs. This uneven distribution suggests that different bHLH lineages have experienced different degrees of retention or expansion in pear. The pan-genome category composition also varied among clades. Core OGGs were broadly distributed across the tree, with relatively high numbers in Clade N, Clade H, Clade C, Clade L, and Clade M. Dispensable OGGs were also detected in multiple clades, especially Clade A, Clade G, Clade H, Clade B, Clade J, and Clade L, suggesting that clade-specific presence/absence variation contributed to bHLH family diversification among pear assemblies. These results indicate that the pear bHLH family is broadly conserved across major evolutionary lineages, with moderate diversification in several clades. Based on characterized *Arabidopsis thaliana* homologs, several major pear bHLH clades may be associated with flavonoid metabolism, light and hormone responses, iron homeostasis, epidermal development, and stress responses.

### 2.4. Genome-Wide Duplication and Selection Patterns Across Pear Genomes

**Figure 4:**
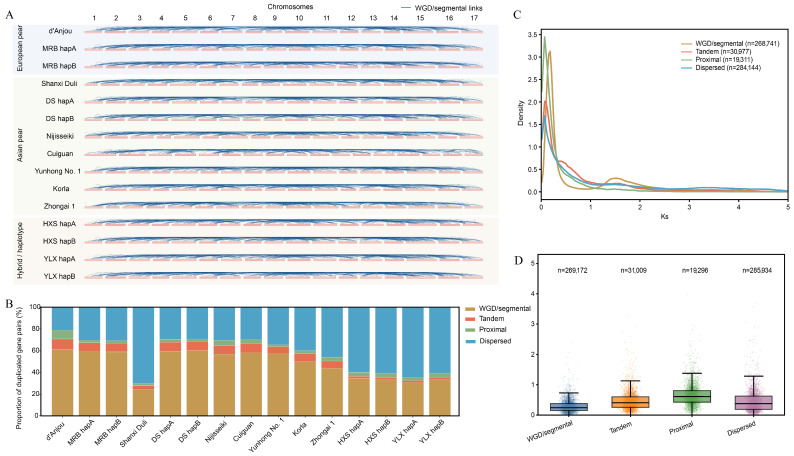
Genome-wide duplication and selection patterns across 15 pear genome assemblies. (**A**) Genome-wide WGD/segmental duplication landscape across 15 pear genome assemblies. Each row represents one genome assembly, and blue arcs indicate representative WGD/segmental duplicated gene pairs. Assemblies are grouped as European pear, Asian pear, and hybrid/haplotype genomes. Chromosome or chromosome-like blocks are shown in light red. (**B**) Proportional composition of genome-wide duplicated gene pairs classified as WGD/segmental, tandem, proximal, and dispersed duplicates in each assembly. (**C**) Ks density distribution of genome-wide duplicated gene pairs among different duplication types. (**D**) Ka/Ks distribution of genome-wide duplicated gene pairs classified by duplication type. Boxes indicate the interquartile range, horizontal lines indicate medians, whiskers show the data range excluding extreme outliers, and dots represent individual duplicated gene pairs.

To provide an evolutionary background for subsequent PbrbHLH family analysis, genome-wide duplicated gene pairs were classified and analyzed across the 15 pear genome assemblies. WGD/segmental duplicated pairs were widely distributed across chromosomes or chromosome-like blocks in all assemblies, indicating that large-scale duplication is a common feature of pear genome evolution (Figure 4A). The proportional composition of duplicated gene pairs varied among assemblies, but WGD/segmental and dispersed duplicates represented the major duplication categories in most genomes, whereas tandem and proximal duplicates accounted for smaller proportions (Figure 4B). Because duplicated-pair numbers may be influenced by assembly quality and gene annotation scale, proportional comparison was used to describe the overall duplication-type composition among genomes. Ks density analysis showed distinct distribution patterns among duplication types (Figure 4C). Most duplicated gene pairs were concentrated at low Ks values, while WGD/segmental pairs also showed a broader distribution with an additional peak at higher Ks values, consistent with the contribution of relatively ancient large-scale duplication events. Ka/Ks analyses showed that most genome-wide duplicated gene pairs had Ka/Ks values below 1 across all duplication types (Figure 4D), suggesting that purifying selection was the dominant evolutionary force after duplication. Compared with WGD/segmental pairs, tandem and proximal duplicated pairs showed relatively broader Ka/Ks distributions, implying that some locally duplicated genes may have experienced more relaxed selective constraints. Together, these results establish a genome-wide duplication and selection background for interpreting the expansion and evolutionary conservation of *PbrbHLH* genes.

### 2.5. Duplication Patterns and Selection Pressure of Pear bHLH Genes

**Figure 5:**
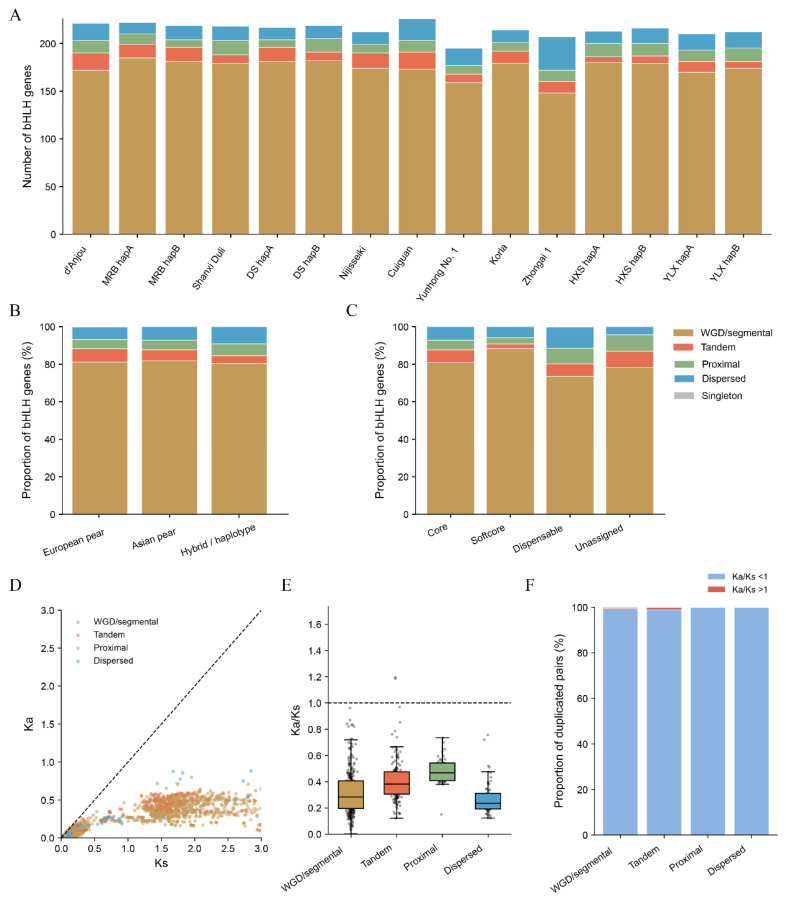
Duplication patterns and selection pressure of pear *bHLH* genes. (**A**) Duplication type composition of *bHLH* genes across 15 pear genome assemblies. Different colors indicate WGD/segmental, tandem, proximal, dispersed, and singleton duplication types. (**B**) Proportional distribution of *bHLH* duplication types among European pear, Asian pear, and hybrid/haplotype groups. (**C**) Proportional distribution of *bHLH* duplication types among different OGG classes, including core, softcore, dispensable, and unassigned groups. (**D**) Relationship between Ka and Ks values of duplicated *bHLH* gene pairs from different duplication types. The dashed diagonal line represents Ka = Ks. (**E**) Ka/Ks distribution of duplicated *bHLH* gene pairs among different duplication types. The dashed horizontal line indicates Ka/Ks = 1. (**F**) Proportion of duplicated *bHLH* gene pairs with Ka/Ks < 1 and Ka/Ks > 1 among different duplication types.

To further investigate the evolutionary expansion of pear *bHLH* genes, duplication types were classified for PbrbHLHs across the 15 pear genome assemblies. The number and proportion of *bHLH* genes assigned to different duplication types varied among assemblies, but WGD/segmental duplication represented the predominant duplication type in most genomes (Figure 5A and Table S4). This pattern indicates that large-scale duplication events contributed substantially to the expansion and retention of the pear bHLH family. When the assemblies were grouped into European pear, Asian pear, and hybrid/haplotype groups, broadly similar duplication-type compositions were observed, although the relative proportions of WGD/segmental, dispersed, and singleton genes differed among groups (Figure 5B). Further integration with OGG classification showed that core and softcore *bHLH* genes were mainly associated with WGD/segmental duplication, whereas dispensable and unassigned genes contained relatively higher proportions of dispersed or singleton genes (Figure 5C). These results suggest that conserved bHLHs were more likely retained after large-scale duplication, while variable bHLHs may have experienced more lineage-specific retention or loss. To evaluate selective pressure on duplicated *bHLH* genes, Ka, Ks, and Ka/Ks values were calculated for duplicated *bHLH* gene pairs. After filtering pairs with invalid or failed estimates, 2418 duplicated *bHLH* gene pairs with valid Ka/Ks values were retained for downstream analysis. The Ka–Ks scatter plot showed that most duplicated *bHLH* gene pairs were located below the Ka = Ks reference line, indicating that Ka values were generally lower than Ks values (Figure 5D). Consistently, the Ka/Ks distributions among different duplication types were mostly below 1 (Figure 5E), suggesting that duplicated *bHLH* genes were predominantly subjected to purifying selection. The proportion analysis further confirmed that most duplicated *bHLH* gene pairs had Ka/Ks < 1 across WGD/segmental, tandem, proximal, and dispersed duplication types (Figure 5F). A small subset of duplicated pairs showed Ka/Ks > 1, suggesting possible accelerated divergence or relaxed selective constraints after duplication. Overall, these results indicate that WGD/segmental duplication played a major role in bHLH family expansion, while purifying selection contributed to the evolutionary conservation of duplicated *bHLH* genes in pear.

### 2.6. Expression Profiling Identified Fruit-Development-Associated PbrbHLH Candidates

**Figure 6:**
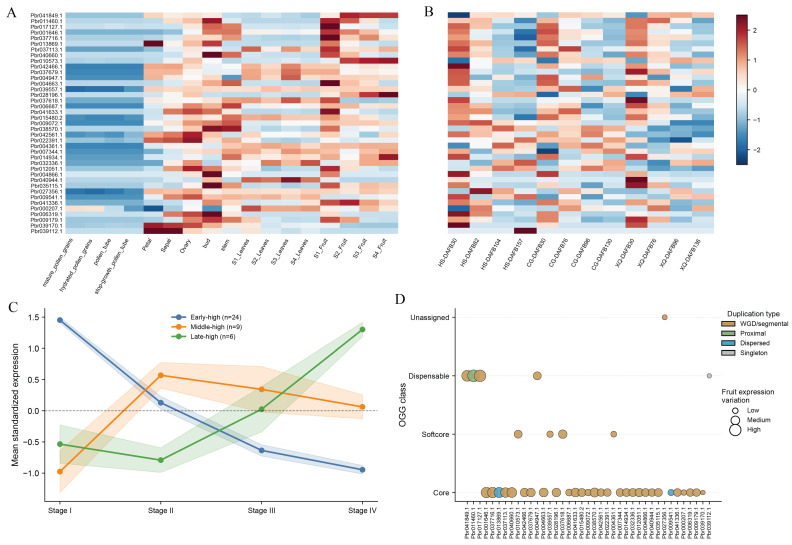
Expression profiling and candidate screening of fruit-development-associated *PbrbHLH* genes. (**A**) Expression heatmap of selected *PbrbHLH* genes across 17 tissues of the pear. (**B**) Expression heatmap of selected *PbrbHLH* genes during fruit development in three pear cultivars. HS, CG, and XQ represent ‘Hosui’, ‘Cuiguan’, and ‘Xueqing’ pears, respectively. DAFB indicates days after full bloom. Expression values were log2-transformed and row-normalized as Z-scores. (**C**) Expression trend clustering of selected *PbrbHLHs* during fruit development. The four developmental stages were defined according to the sequential sampling points within each cultivar. Lines represent the mean standardized expression values of genes in each trend group, and shaded areas indicate the variation among genes within each group. (**D**) Integrated feature plot of selected PbrbHLH candidates. The *y*-axis indicates OGG class, dot color represents duplication type, and dot size indicates fruit expression variation.

Because matched fruit-development transcriptomes were not available for all 15 pear genome assemblies, available pear transcriptome datasets were used as an independent expression layer for transcriptome-guided candidate prioritization. To further explore the potential roles of PbrbHLHs in pear fruit development, expression profiles were extracted from 17-tissue and fruit-development transcriptome datasets. Among the identified *PbrbHLHs*, 176 genes were successfully matched to both expression matrices. Candidate genes were then prioritized mainly according to their expression intensity, dynamic range, and fold change during fruit development, together with OGG classification and tissue expression variation. Based on this screening strategy, 39 representative *PbrbHLHs* were selected for visualization and further analysis (Table S5). The selected *PbrbHLHs* showed diverse expression patterns across tissues and fruit developmental stages (Figure 6A,B and Table S5). Several genes displayed relatively high expression in fruit or reproductive tissues, whereas others showed broader or more tissue-specific expression patterns. In the fruit-development dataset, the selected *PbrbHLHs* could be grouped into distinct temporal expression trends, including early–high, middle–high, and late–high patterns (Figure 6C). The early–high group accounted for a large proportion of the selected genes, suggesting that many candidate *PbrbHLHs* may be involved in early fruit developmental processes. A smaller number of genes showed increased expression at later stages, implying possible roles in later fruit development or maturation-related processes. Integration with pan-genome annotation showed that the selected candidates were mainly derived from core and softcore OGG classes, while several dispensable *PbrbHLHs* also showed strong fruit-development-associated expression variation (Figure 6D). Most candidates were associated with WGD/segmental duplication, consistent with the dominant contribution of this duplication type to the expansion of the pear bHLH family. These results suggest that both conserved and variable *PbrbHLHs* may contribute to fruit-development-related regulatory divergence in pear.

### 2.7. Candidate PbrMYB–PbrbHLH Co-Expression Modules Identified from Available Pear Fruit-Development Transcriptomes

**Figure 7:**
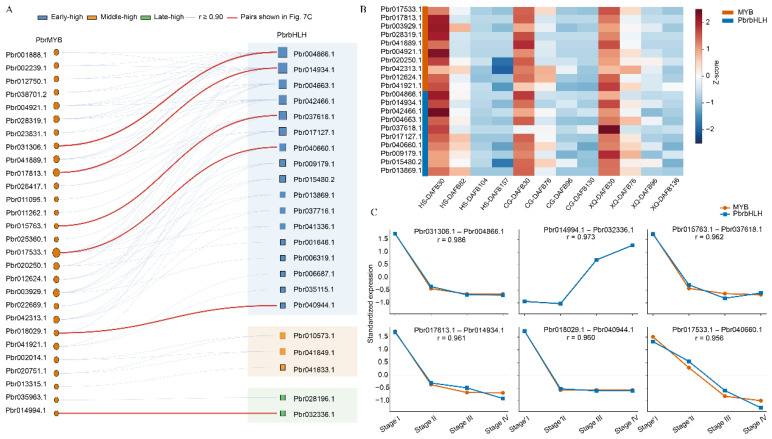
Candidate PbrMYB–PbrbHLH co-expression network identified from available pear fruit-development transcriptomes. (**A**) Bipartite co-expression network between PbrMYBs and candidate PbrbHLHs. Pearson correlation coefficients were calculated using log2-transformed RPKM values across 12 fruit developmental samples from three pear cultivars. Only highly positive correlations with r ≥ 0.90 are shown. *PbrMYB* and *PbrbHLH* genes are arranged on the left and right sides, respectively. PbrbHLH nodes are grouped according to their fruit-development expression trends, including early–high, middle–high, and late–high. Pale blue lines indicate high-confidence positive co-expression edges, and red lines indicate the representative pairs shown in panel C. (**B**) Expression heatmap of hub *PbrMYBs* and *PbrbHLHs* identified from the co-expression network. Expression values were log2-transformed and row-standardized. Orange and blue side bars indicate *PbrMYB* and *PbrbHLH* genes, respectively. (**C**) Expression trends in six representative PbrMYB–PbrbHLH pairs during fruit development. Expression values were averaged across the three cultivars at each developmental stage and then standardized. Pearson correlation coefficients are shown for each pair. HS, ‘Hosui’; CG, ‘Cuiguan’; XQ, ‘Xueqing’; DAFB, days after full bloom.

To further explore potential regulatory relationships associated with pear fruit development, co-expression analysis was performed between the identified *PbrMYB* genes and the 39 candidate *PbrbHLHs* selected from the fruit-development expression profiling. The fruit-development transcriptome dataset included 12 samples from three pear cultivars: HS, CG, and XQ. Among these cultivars, CG was included in the 15 genome assemblies used for pan-genome analysis, whereas HS and XQ were used as additional available fruit-development transcriptome datasets with comparable developmental stages. Therefore, this analysis was designed for transcriptome-guided candidate prioritization and cross-cultivar expression support, rather than for constructing a pan-genome-wide expression network across all 15 assemblies. Pearson correlation coefficients were calculated across the 12 fruit-development samples. Using a high-confidence threshold of r ≥ 0.90, 73 positive PbrMYB–PbrbHLH co-expression pairs were retained and used to construct a bipartite network (Figure 7A; Table S6). The network contained a subset of strongly connected *PbrMYBs* and *PbrbHLHs* rather than all candidate genes, indicating that only part of the PbrbHLH family showed highly coordinated expression with *MYB* genes during fruit development. Most connected PbrbHLHs belonged to the early–high expression group, while a smaller number were assigned to the middle–high and late–high groups (Figure 7A). This pattern suggests that early fruit development may represent an important stage for coordinated MYB–PbrbHLH transcriptional regulation. Hub genes from the network were further examined using the fruit-development expression matrix. The heatmap showed that these hub *PbrMYBs* and *PbrbHLHs* displayed clear stage- and cultivar-associated expression differences (Figure 7B). Several hub genes showed higher expression at early developmental stages, whereas others showed increased expression at later stages, supporting their potential involvement in distinct phases of pear fruit development. Six representative PbrMYB–PbrbHLH pairs were selected from the high-confidence network to visualize their expression trends. These pairs showed highly similar temporal expression patterns across the four developmental stages, consistent with their strong positive correlations (Figure 7C). For example, *Pbr031306.1*–*Pbr004866.1* and *Pbr015763.1*–*Pbr037618.1* showed early–high expression patterns, whereas *Pbr014994.1*–*Pbr032336.1* displayed a late-increasing trend. These results highlight several candidate MYB–PbrbHLH co-expression modules identified from the available pear fruit-development transcriptomes of HS, CG, and XQ. Because these transcriptome datasets were not matched with all 15 genome assemblies used for pan-genome analysis, the identified modules should be interpreted as transcriptome-guided candidate associations rather than pan-genome-wide regulatory modules across pear germplasm.

## 3. Discussion

Single-reference-based gene family analyses have been widely used in plants, but they may underestimate gene presence/absence variation, copy-number variation, and accession-specific members within genetically diverse species. Recent pan-genome-based studies of other plant gene families, such as the O-methyltransferase family in tomato, have further shown that pan-genome resources are useful for revealing gene-family evolutionary dynamics and functional divergence [24]. Previous pan-genome studies have emphasized that multiple genome assemblies can provide a more complete representation of species-level genetic diversity than a single reference genome, especially for gene families affected by duplication, retention, and loss [25,26]. This view is also supported by a recent barley study, in which pangenome and pantranscriptome resources were used to improve the characterization of the bHLH family and to distinguish conserved and variable gene members across accessions [4]. In pear, the availability of multiple genome assemblies, including haplotype-resolved genomes and a pangenome graph, has provided a stronger genomic basis for studying gene diversity among Asian pears, European pears, and hybrid cultivars [27]. Consistent with this idea, our analysis across 15 pear genome assemblies allowed PbrbHLHs to be characterized within a broader pan-genome framework rather than from a single reference genome alone (Figure 1).

The OGG-based classification divided PbrbHLHs into core, softcore, and dispensable groups, with a small number of unassigned genes (Figure 2). Core and softcore PbrbHLHs may represent a relatively stable regulatory backbone maintained across pear genomes, whereas dispensable and unassigned members may reflect lineage-specific retention, gene loss, or haplotype-level variation. Similar interpretations have been proposed in pan-genome-based gene family studies, where variable members are often considered important for understanding genome diversification and potential trait-associated differences among accessions [3,4]. Also taking into account the bHLH protein tree (Figure 3), these results suggest that pear bHLH evolution involves both conserved family structure and genome-specific diversification, providing a basis for subsequent analyses of gene duplication, selection pressure, and fruit-development-associated expression divergence.

Gene duplication is a major driver of plant gene family expansion, providing genetic material for dosage retention, subfunctionalization, and neofunctionalization [28,29]. In pear, ancient whole-genome duplication and subsequent segmental duplication have been considered important forces shaping genome evolution and gene-family diversification [28,30]. Consistent with this general pattern, our genome-wide duplication analysis showed that WGD/segmental duplication was a major component of the pear duplication landscape (Figure 4). This broader genomic background helps explain why many *PbrbHLH* members were retained as duplicated genes rather than appearing as isolated family-specific events. For transcription factor families, duplicated copies are often retained because regulatory genes are dosage sensitive and participate in complex interaction networks [29]. In this study, WGD/segmental duplication contributed substantially to the conserved PbrbHLH repertoire, especially among core and softcore members (Figure 5). This suggests that large-scale duplication may have helped maintain a stable bHLH regulatory backbone in pear. In contrast, variable *PbrbHLHs* may have been more affected by dispersed duplication, gene loss, or lineage-specific retention. Most duplicated *PbrbHLH* gene pairs show Ka/Ks values below 1; this would further indicate that purifying selection has acted on duplicated *PbrbHLH* genes after duplication, supporting their functional constraint. A small number of pairs with elevated Ka/Ks values may represent candidates for relaxed selection or functional divergence, but these cases require further experimental validation.

Gene-family expansion does not necessarily imply functional divergence, and transcriptome evidence is often needed to prioritize biologically relevant candidates. bHLH transcription factors have been reported to participate in multiple fruit-related processes, including pigment accumulation, flavonoid metabolism, hormone signaling, ethylene biosynthesis, and ripening regulation [7,8]. In pear, previous transcriptome studies have shown that fruit development and ripening involve extensive transcriptional reprogramming, and several transcription factor families, including *MYB*, *bHLH*, *ERF*, and *MADS-box* genes, have been associated with fruit quality traits [19,21,31]. Therefore, integrating pan-genome-based family analysis with expression profiles is useful for distinguishing broadly retained genes from candidates more likely to be involved in fruit development. In this study, 17-tissue expression data and fruit-development transcriptomes from three pear cultivars were used to screen candidate PbrbHLHs with fruit-related expression variation (Figure 6). The selected candidates showed distinct temporal patterns, including early-, middle-, and late-stage high expression, suggesting that different PbrbHLH members may be deployed at different phases of fruit development. The early–high, middle–high, and late–high expression groups suggest that different PbrbHLHs may function at different stages of pear fruit development. Early–high candidates may be related to early fruit growth and texture formation, whereas middle–high or late–high candidates may be associated with ripening-related processes, including ethylene response, pigmentation, secondary metabolism, and sugar/acid accumulation. However, these functional links remain putative and require further experimental validation. This stage-specific expression divergence is consistent with the functional diversity of plant bHLHs reported in other species, where different members often regulate distinct developmental or metabolic processes [4]. In our previous work, *PbbHLH164* (*Pbr011456.1*) was classified as a softcore member in OG0000139 and was retained as a fruit-development-associated candidate in this study [21]. This consistency supports the reliability of the pan-genome and transcriptome-guided screening strategy. The PbrbHLHs identified here provide useful targets for future studies on pear fruit development, especially genes showing both conserved evolutionary features and strong developmental expression dynamics.

bHLH proteins often act together with other transcription factors. The MYB–bHLH–WD40 complex is a classical regulatory module involved in anthocyanin, proanthocyanidin, and flavonoid metabolism, as well as several developmental processes [10,11]. Structural studies have also shown that MYB and bHLH proteins can form specific complexes through defined partner-selection mechanisms [12], supporting the potential biological relevance of MYB–bHLH combinations. In this study, co-expression analysis identified several highly correlated PbrMYB–PbrbHLH pairs during pear fruit development (Figure 7). These pairs showed similar temporal expression patterns, suggesting that they may participate in related developmental processes. For example, the candidate pair involving the *MYB* gene *Pbr014994.1* showed coordinated expression patterns, indicating a potential regulatory link between MYB and bHLH members during pear fruit development. However, it should be noted that the fruit-development transcriptomes used for co-expression analysis were derived from HS, CG, and XQ, whereas only CG was included among the 15 genome assemblies used for pan-genome analysis. Therefore, the PbrMYB–PbrbHLH network should not be interpreted as a pan-genome-wide regulatory network across all 15 pear assemblies. Instead, it represents transcriptome-guided candidate co-expression associations supported by the available fruit-development expression datasets. Matched genome and fruit-development transcriptome datasets from more pear accessions will be required to determine whether these candidate modules are conserved across broader pear germplasm. These MYB–bHLH relationships are based on co-expression analysis and should be regarded as candidate modules. Further qRT-PCR, protein–protein interaction assays, and transient expression analyses will be required to validate their regulatory roles.

## 4. Materials and Methods

### 4.1. Identification of bHLH Genes in Pear Genome Assemblies

Protein sequences from 15 pear genome assemblies were used for genome-wide identification of bHLH family members. These assemblies included *Pyrus pyrifolia* ‘Cuiguan’ [32], *P. betulifolia* ‘Shanxi Duli’ [33], *P. pyrifolia* ‘Nijisseiki’, *P. ussuriensis* × *P. communis* ‘Zhongai 1’ [34], *P. communis* ‘d’Anjou’ [35], *P. sinkiangensis* ‘Korla’ [36], and *P. pyrifolia* ‘Yunhong No. 1’ [37]. In addition, haplotype-resolved genome assemblies were included for *P. sinkiangensis* × *P. bretschneideri* ‘Hongxiangsu’, including ‘Hongxiangsu’ haplotype A (KEL haplotype) and haplotype B (EL haplotype), and for *P. sinkiangensis* × *P. bretschneideri* ‘Yuluxiang’, including ‘Yuluxiang’ haplotype A (KEL haplotype) and haplotype B (XH haplotype) [38]. The T2T haplotype-resolved assemblies of *P. bretschneideri* ‘Dangshansuli’ (DS_hapA and DS_hapB) and *P. communis* ‘Max Red Bartlett’ (MRB_hapA and MRB_hapB) were also included [39]. The previously reported old ‘Dangshansuli’ [5] Pbr gene set was not treated as one of the 15 assemblies for pan-genome analysis but was used as a reference for Pbr ID correspondence and comparisons with published pear *bHLH* genes. Basic information for these genomes is summarized in Table S1. The previously reported *PbrbHLH* genes [22] from the ‘Dangshansuli’ genome were not included as one of the fifteen assemblies for pan-genome analysis, but they were used as a reference set to calibrate the identification pipeline and to facilitate subsequent correspondence with conventional Pbr gene IDs. Candidate bHLH proteins were identified using a combined HMMER–BLASTP strategy. First, the hidden Markov model profile of the bHLH domain (PF00010) was used as a query to search against each pear proteome using HMMER (v 3.4) with a relaxed threshold. In parallel, previously reported PbrbHLH protein sequences from ‘Dangshansuli’ were used as queries for BLASTP (v 2.17.0+) searches against each pear proteome to recover additional potential bHLH members that might be missed by HMMER alone. Candidate proteins identified by either approach were merged, and redundant sequences were removed. The merged candidates were then subjected to a second PF00010 domain confirmation using HMMER. Only proteins containing a detectable bHLH domain with an aligned domain length of at least 30 amino acids were retained for downstream analyses. Candidates without a confirmed bHLH domain, with excessively short domain alignments, or representing obvious non-bHLH matches were discarded. Using this pipeline, the final bHLH candidate set was obtained for each of the 15 pear genome assemblies. The old ‘Dangshansuli’ PbrbHLH dataset was also screened using the same criteria to evaluate consistency with previously reported pear *bHLH* genes, and the resulting Pbr ID correspondence was used for later annotation of pear bHLH orthogroups.

### 4.2. Orthogroup Clustering and Pan-Genome Classification

The final bHLH protein sequences from the 15 pear genome assemblies were subjected to orthogroup clustering using OrthoFinder (https://github.com/davidemms/OrthoFinder) (accessed on 20 May 2026). The resulting orthogroups were defined as bHLH OGGs, and the copy number of each OGG in each assembly was obtained from the OrthoFinder output. Based on their presence across the 15 assemblies, OGGs were classified as core OGGs, softcore OGGs, dispensable OGGs, or private OGGs. OGGs present in all 15 assemblies were defined as core, those present in 14 assemblies as softcore, those present in 2–13 assemblies as dispensable, and those present in only one assembly as private. *bHLH* genes that were not assigned to any OGG were retained as unassigned genes. For the pan/core curve, an OGG presence/absence matrix was constructed. For each genome number, different combinations of assemblies were used to calculate the number of pangenome and core OGGs. Pangenome OGGs were defined as OGGs present in at least one assembly in a given combination, whereas core OGGs were defined as OGGs present in all assemblies in that combination. The mean and standard deviation were calculated and plotted. Gene-level OGG composition and copy-number variation were visualized using custom Python (v 3.10.20) scripts. Copy numbers of three or more were grouped as ≥3 in the heatmap.

### 4.3. Evolutionary Analysis

To infer the evolutionary relationships of pear *bHLH* genes, representative protein sequences were selected from each bHLH orthogroup. The longest protein sequence within each orthogroup was used as the representative sequence. In addition, 160 *Arabidopsis* bHLH protein sequences retrieved from the TAIR10 primary transcript annotation were included as reference sequences for evolutionary classification. Multiple sequence alignment was performed using MAFFT (https://mafft.cbrc.jp/alignment/software/) (accessed on 20 May 2026), and poorly aligned regions were trimmed using trimAl (http://trimal.cgenomics.org/). A maximum-likelihood evolutionary tree was constructed using IQ-TREE (http://www.iqtree.org/) (accessed on 20 May 2026) with the LG+G4 model and 1000 ultrafast bootstrap replicates. The resulting tree was visualized and annotated using iTOL (https://itol.embl.de/) (accessed on 20 May 2026).

### 4.4. Gene Duplication and Ka/Ks Analysis

Gene duplication types were classified for each pear genome assembly using an MCScanX-based workflow. Protein sequences from each assembly were compared by all-versus-all DIAMOND BLASTP, and the resulting similarity files, together with gene-position information extracted from GFF3 annotations, were used as inputs for MCScanX (https://github.com/wyp1125/MCScanX) (accessed on 20 May 2026). The duplicate_gene_classifier module was used to classify genes into singleton, dispersed, proximal, tandem, and WGD/segmental duplicates. Transposed duplicates were not treated as an independent category in this workflow; therefore, all duplication-type comparisons were interpreted within the same MCScanX-based classification framework. Duplicated gene pairs were extracted from the MCScanX outputs and retained as the genome-wide background dataset. PbrbHLH-related duplicated pairs were obtained by matching the genome-wide duplicated-pair table with the identified *PbrbHLH* genes, ensuring that bHLH-specific duplication patterns were derived from the same background analysis. The duplication results were then integrated with OGG classification and evolutionary clade annotation to summarize PbrbHLH duplication patterns across assemblies, pear groups, and OGG classes. For Ka/Ks analysis, protein and CDS sequences of duplicated gene pairs were extracted. Protein alignments were generated using MAFFT and converted into codon alignments using PAL2NAL (https://bio.tools/pal2nal) (accessed on 20 May 2026). Ka, Ks, and Ka/Ks values were calculated using the yn00 program in PAML (https://github.com/abacus-gene/paml) (accessed on 20 May 2026). Pairs with missing sequences, failed alignments, undefined Ks values, zero Ks values, or invalid Ka/Ks estimates were excluded. Genome-wide duplicated pairs were used to describe the overall evolutionary background, whereas duplicated *PbrbHLH* gene pairs were used for family-specific evolutionary analysis.

### 4.5. Visualization of Duplication and Ka/Ks Patterns

The duplication-type distribution of *bHLH* genes was summarized at the assembly, pear group, and OGG-class levels. Stacked bar plots were used to show the number and proportion of *bHLH* genes assigned to each duplication type. For Ka/Ks analysis, genome-wide duplicated gene pairs were first summarized to provide an overall view of duplication-associated evolutionary patterns. The bHLH-related duplicated pairs were then analyzed separately. Ka, Ks, and Ka/Ks distributions were visualized using scatter plots, boxplots, and proportional bar plots. In Ka–Ks scatter plots, duplicated pairs were colored according to duplication type, and a reference line of Ka = Ks was added to indicate Ka/Ks = 1. The proportions of duplicated pairs with Ka/Ks < 1 and Ka/Ks > 1 were further calculated for different duplication types. All statistical summaries and visualizations were generated using custom Python scripts.

### 4.6. Expression Profiling and Candidate PbrbHLH Screening

Expression profiles of *PbrbHLH* genes were analyzed using two transcriptome datasets. The 17-tissue expression dataset of ‘Dangshansuli’ pear was obtained from PearMODB (http://pcwgdb.njau.edu.cn/) (accessed on 20 May 2026) [27]. The fruit-development expression dataset was derived from our previously published RNA-seq data of three pear cultivars during fruit development [15]. In the fruit-development dataset, HS, CG, and XQ represent ‘Hosui’, ‘Cuiguan’, and ‘Xueqing’ pears, respectively. Samples were collected at four developmental stages (Stage I included HS-DAFB30, CG-DAFB30, and XQ-DAFB30; Stage II included HS-DAFB82, CG-DAFB76, and XQ-DAFB76; Stage III included HS-DAFB104, CG-DAFB96, and XQ-DAFB96; Stage IV included HS-DAFB157, CG-DAFB130, and XQ-DAFB136). DAFB indicates days after full bloom. *PbrbHLH* genes were matched to the expression matrices according to their corresponding ‘Dangshansuli’ reference gene IDs. Expression values were transformed as log2(RPKM + 1), and row-normalized Z-scores were used for heatmap visualization. Candidate *PbrbHLHs* were screened from genes that could be matched to both the 17-tissue and fruit-development expression matrices. Screening mainly considered fruit-development expression features, including maximum expression level, dynamic range across developmental stages, and maximum/minimum fold change. The OGG class was also considered, with core and softcore genes given higher priority. To avoid missing tissue-specific candidates, genes with strong expression variation across the 17 tissues were also retained. The final candidate set was used for expression heatmaps, trend clustering, and integrated feature visualization. For fruit-development trend analysis, the expression values of each candidate *PbrbHLH* were averaged across the three cultivars at each developmental stage. The averaged expression profiles were then standardized across the four stages. Candidate genes were grouped according to the stage showing the highest standardized expression value, including early–high, middle–high, and late–high expression patterns.

### 4.7. PbrMYB–PbrbHLH Co-Expression Analysis

The pan-genome and transcriptome analyses were treated as two complementary layers. The 15 pear genome assemblies were used to characterize *bHLH* gene repertoire, OGG classification, duplication patterns, and selection pressure, whereas the fruit-development transcriptomes were used to prioritize candidate PbrbHLHs and MYB–bHLH co-expression associations. Because matched fruit-development transcriptomes were not available for all 15 genome assemblies, three available pear fruit-development transcriptome datasets, HS, CG, and XQ, were used for expression-based candidate screening. CG was included in the 15 genome assemblies, while HS and XQ provided additional cross-cultivar expression support. To identify candidate MYB–bHLH regulatory modules associated with pear fruit development, co-expression analysis was performed using the same fruit-development expression matrix. A total of 185 *PbrMYB* genes from our previous MYB family dataset [40] and the 39 candidate PbrbHLHs selected from expression profiling were used for correlation analysis. Expression values were transformed as log2(RPKM + 1), and Pearson correlation coefficients were calculated for each PbrMYB–PbrbHLH pair across the 12 fruit-development samples. Gene pairs with high positive correlations were retained to construct the candidate PbrMYB–PbrbHLH co-expression network. A stringent Pearson correlation threshold of r ≥ 0.90 was used to retain strongly positive candidate PbrMYB–PbrbHLH co-expression pairs. Hub genes were identified according to node degree in the network. Six representative PbrMYB–PbrbHLH pairs were selected from the high-confidence network based on high Pearson correlation coefficients while avoiding repeated *PbrMYB* or *PbrbHLH* genes where possible. For cross-cultivar consistency analysis, the expression trends in the six representative PbrMYB–PbrbHLH pairs were examined separately in HS, CG, and XQ across the four developmental stages. Their expression trends were averaged across the three cultivars at each developmental stage and then standardized for visualization. The resulting network represents co-expression-based candidate associations rather than experimentally validated physical interactions.

## 5. Conclusions

In this study, we established a genome-wide duplication and Ka/Ks framework across 15 pear genome assemblies and used it to analyze the evolutionary diversification of the *bHLH* gene family. A total of 3222 *bHLH* genes were identified, including both conserved and variable members, indicating that the pear bHLH family has been shaped by genome conservation and diversification. Duplication analysis showed that WGD/segmental duplication contributed to *bHLH* expansion, while most duplicated genes were constrained by purifying selection. Transcriptome integration further identified 39 fruit-development-associated PbrbHLHs, and co-expression analysis revealed candidate PbrMYB–PbrbHLH modules. These results provide an evolutionary framework and candidate regulatory modules for future studies of pear fruit development.

## Data Availability

The original contributions presented in this study are included in the article/Supplementary Material. Further inquiries can be directed to the corresponding author.

## Supplementary Materials

The following supporting information can be downloaded at https://www.mdpi.com/article/10.3390/plants15131961/s1. Table S1: Profile of 15 pear genome assemblies and the ‘Dangshansuli’ pear genomes. Table S2: Detailed information and Pbr ID correspondence of *bHLH* genes identified from 15 pear genome assemblies. Table S3: Summary of bHLH orthogroups, clade classification, and assembly distribution across 15 pear genome assemblies. Table S4: Ka, Ks, and Ka/Ks values of duplicated *bHLH* gene pairs in pear genomes. Table S5: Expression matrix of the 39 candidate *PbrbHLH* genes across 17 tissues and 3 pear cultivars during fruit development. Table S6: High-confidence MYB–PbrbHLH co-expression pairs during pear fruit development.

## Abbreviations

bHLH	Basic helix–loop–helix
MYB	Myeloblastosis
WD40	Tryptophan-aspartic acid repeat protein
MBW	MYB–bHLH–WD40
TF	Transcription factor
OGG	Orthologous gene group
WGD	Whole-genome duplication
Ka	Nonsynonymous substitution rate
Ks	Synonymous substitution rate
Ka/Ks	Ratio of nonsynonymous to synonymous substitution rates
RPKM	Reads per kilobase of transcript per million mapped reads
DAFB	Days after full bloom
HMM	Hidden Markov model
